# The PacBio Full-Length Transcriptome of the Tea Aphid as a Reference Resource

**DOI:** 10.3389/fgene.2020.558394

**Published:** 2020-11-12

**Authors:** Feng Hong, Si-Hua Mo, Xing-Yu Lin, Jinzhi Niu, Jian Yin, Dong Wei

**Affiliations:** ^1^College of Agriculture, Xinyang Agriculture and Forestry University, Xinyang, China; ^2^Chongqing Key Laboratory of Entomology and Pest Control Engineering, College of Plant Protection, Southwest University, Chongqing, China; ^3^State Cultivation Base of Crop Stress Biology for Southern Mountainous Land, Academy of Agricultural Sciences, Southwest University, Chongqing, China

**Keywords:** Iso-seq, SMRT sequencing, *Aphis aurantii*, reference resource, long non-coding RNA, simple sequence repeat

## Abstract

The tea aphid, *Aphis aurantii*, has become one of the destructive pests in tea plantations in the tropics and subtropics. Very few functional studies have so far focused on the developmental and reproductive biology at a molecular level, because of the lack of comprehensive genetic information. Full-length transcriptomes represent a very highly efficient approach to obtain reference gene sequences in non-model insects. In the present study, the transcriptome of *A. aurantii* was comprehensively sequenced using PacBio Iso-Seq technology. A total of 46.8 Gb nucleotides and 15,938 non-redundant full-length transcripts were obtained, 13,498 (84.69%) of which were annotated into seven databases. Of these transcripts, 2,029 alternative splicing events and 15,223 simple sequence repeats were detected. Among these transcripts, 4,571 (28.68%) and 11,367 (71.32%) were long non-coding RNAs (lncRNAs) and protein-coding genes, respectively. Five hundred and ninety transcription factors were detected. The first full-length transcriptome represents a significant increase in the known genetic information of *A. aurantii*. It will assist the future functional study of genes involved in its development and reproduction.

## Introduction

The tea aphid, *Aphis aurantii* (Boyer de Fonscolombe), was first described in 1841 in citrus fruit in France, but has since expanded to many other countries in the tropics and subtropics, even to a cold northern region (Piron et al., 2019). It is prevalent in tea plantations and has become the most destructive pest (Han et al., 2012). *A. aurantii* is a polyphagous aphid, it is also known as black citrus aphid, destroying fruit in citrus orchards (Wang and Tsai, 2001). It can damage the tips of shoots or new fresh leaves by feeding on their phloem sap and injecting its saliva into the plants which causes phytotoxicity (Sarac et al., 2015). This pest has high fecundity and reproduces quickly in large numbers, making it difficult to control in tea and citrus orchards (Wang and Tsai, 2001). Similar to other aphid vectors transmitting plant virus diseases (Pinheiro et al., 2017; MacKenzie et al., 2018), *A. aurantii* also transmits viruses, resulting in reduced production due to smaller trees and fruits (Rao and Capoor, 1976; Allan, 1980). Moreover, *A. aurantii* secretes honeydew on which sooty molds frequently grow, decreasing photosynthetic activity (Piron et al., 2019).

Transcriptome represents a highly efficient approach to obtaining the reference gene sequences of non-model insects. Quantitative and qualitative transcriptomes reflect comprehensive physiological processes at a molecular level. This technique has been frequently used in non-model insects without available genomes (Morandin et al., 2018; Zhang et al., 2018), in addition to insects where the genomes are available, e.g., *Zeugodacus cucurbitae* (Wei et al., 2020). Illumina sequencing based on next-generation sequencing (NGS) technology has limitations to the complete and accurate assembly of transcripts, recognition of alternative splicing (AS) isoforms or homologous genes families. The full-length transcriptome sequencing approach is an alternative method for obtaining complete and accurate transcripts (Wang X. et al., 2019). Currently, full-length transcriptome sequencing mostly uses PacBio single-molecular real-time (SMRT) sequencing technology with long-read sequencing characteristics (Rhoads and Au, 2015). It minimizes low-quality assembly of short reads, and directly produces single complete transcripts, and is advantageous for the identification of gene/isoform. This new technique is well-suited for unsolved problems in genome, transcriptome, and epigenetics research. This technique provides more reliable evidence for the qualitative analysis of AS transcripts and improves genome annotation (Wang X. et al., 2019; Yin et al., 2019).

Very few studies have focused on the functional analysis of the development and reproduction of *A. aurantii*, possibly because of the limited reference sequences. The transcriptomes of an increasing number of species have been deposited and accessible in public databases, such as the National Center of Biotechnology Information (NCBI) Sequence Read Archive (SRA) database, which is the most popular repository of RNA-Seq data. No such data for *A. aurantii* is available in that database, as the majority of the published studies have focused on its ecology or control (Aslam et al., 2015; Zekri et al., 2016; Gholamzadeh-Chitgar and Pourmoradi, 2017; Alizadeh Kafeshani et al., 2018; Li L. et al., 2019). In addition to protein-coding genes, non-coding genes also play important roles in insects, e.g., long non-coding RNAs (lncRNAs), defined as transcripts longer than 200 nucleotides that do not show any protein-coding capability (Legeai and Derrien, 2015). More and more studies focus on the lncRNAs identification and their roles in insects (Legeai and Derrien, 2015; Chen M. Y. et al., 2019; Li S. et al., 2019; Li et al., 2020). While no lncRNAs were identified by far in *A. aurantii*.

In the present study, 4th instar nymph and adult *A. aurantii* were sampled and pooled using the same quantities of total RNA from each for transcriptome sequencing and analysis. Complete long-read transcriptomic sequencing was conducted using the PacBio Sequel platform with the SMRT sequencing method. This full-length transcriptome contributes to the first comprehensive dataset of genetic information for this species, including protein-coding genes, lncRNAs, and microsatellites. The data represent an important reference for future functional studies involving development and reproduction. It will also assist in future evolutionary studies of aphids.

## Materials and Methods

### Insect Collection and RNA Sampling

Adults of the aphid *A. aurantii* were collected from a tea plantation in Xinyang, Henan Province, China, 2019. Stock aphids were maintained in a versatile environmental chamber (MLR-352H-PC, Panasonic, Ehime, Japan) at 25°C, relative humidity of 75 ± 5%, and photoperiod of 14 h light: 10 h darkness. The insects were reared with fresh young tender tea shoots. Four batches of newborn nymphs were collected within 4 h and cultured separately. Wingless 4th instar nymphs were collected on day seven and wingless adults collected on day nine. Two biological replicates were collected at each stage, and each sample contained 20 individuals. The total RNA was isolated from each using TRIzol reagent (Invitrogen, Carlsbad, CA, United States), in accordance with the protocol described previously (Wei et al., 2020). The RNA samples were qualitatively and quantitatively evaluated using 1% agarose gel electrophoresis and a NanoDrop One spectrophotometer (Thermo Fisher Scientific, Madison, WI, United States).

### Library Construction

Prior to library construction, the high-quality RNA samples at each stage were pooled in the same quantities. RNA samples were re-checked using a Qubit fluorometer (Life Technologies, Carlsbad, CA, United States) and a Bioanalyzer 2100 (Agilent Technologies, Palo Alto, CA, United States). The Iso-Seq library was prepared in accordance with standard PacBio Iso-Seq sequencing protocol. Oligo (dT) primers were used to enrich mRNA molecules in each sample. A SMARTer PCR cDNA synthesis kit (Clontech, Palo Alto, CA, United States) was used to reverse-transcribe the complementary strand (cDNA) in accordance with standard protocol and then re-amplified using PCR. All cDNAs were end-repaired, connected with adaptors using a hairpin loop, followed by conversion into blunt ends *via* exonucleases. The quality of the library was re-assessed using an Agilent Bioanalyzer 2100 system. SMRT sequencing was then performed using a PacBio Sequel platform (Novogene, Beijing, China).

### SMRT Sequencing and Preprocessing

The raw data were initially processed using the Iso-seq standard pipeline technique using SMRTlink V7.0 to obtain subreads >50 bp. Circular consensus sequences (CCSs) were generated from the subreads. The CCSs were classified into full-length and non-full-length reads depending on the adapters at the 5′ and 3′ ends, in addition to poly(A) tails at the 3′ ends. Subreads containing adaptors and 3′ poly(A) tails were considered full-length subreads. Non-chimeric full-length subreads were termed full-length non-chimeric (FLNC) sequences. Iterative clustering for error correction (ICE) was conducted to cluster the FLNC subreads based on pairwise alignment and reiterative assignment using the hierarchical algorithm: *n*^∗^log(*n*). The clustered consensus subreads were polished with non-full-length fragments to obtain high-quality FLNC isoforms using Arrow software. NGS RNA-Seq data sequenced using an Illumina NovaSeq platform from wingless adults were downloaded from the NCBI SRA database: SRR11285614, SRR11285615, SRR11285616, and SRR11285617, for mismatche correction. Nucleotide mismatches were corrected in the consensus subreads using LoRDEC V0.7 software. Redundancy in the corrected consensus fragments was removed using CD-Hit V4.6.8 software to obtain full-length final unigenes (Li and Godzik, 2006).

### Prediction and Functional Annotation

All unigenes produced as described above were annotated to the NCBI NR, KOG, SwissProt, and KEGG databases using the BLAST tool and the NCBI NT database by BLASTx (McGinnis and Madden, 2004) using a Diamond V0.8.36 tool. These sequences were also functionally annotated to the Pfam database using HMMER v3.1 software, and to the GO database using Metascape (Zhou et al., 2019). Annotation was confirmed based on the most appropriate match between query transcripts and known sequences from accessible databases.

### Alternative Splicing and Simple Sequence Repeat Detection

The non-redundant full-length transcripts were firstly mapped to UniTransModels using gmap software. Splicing junctions for transcripts mapped to the same UniTransModels were examined; these transcripts with the same splicing junctions were screened out. Collapsed transcripts with different splicing junctions were identified as transcriptional isoforms of UniTransModels. Finally, the AS events were detected using SUPPA^1^ software with default settings.

Simple sequence repeats (SSRs), also known as microsatellites, are short repeating DNA motifs (1–6 bp) arranged in tandem. SSRs are widely distributed throughout the genomes of eukaryotic organisms (Sharma et al., 2007), and were identified in this transcriptome using MISA tool with the default parameters (Thiel et al., 2003).

### Analysis of Gene Type

Coding Potential Calculator 2 (CPC2) (Kang et al., 2017), Coding-Non-Coding Index (CNCI) (Sun et al., 2013), PfamScan (Finn et al., 2016), and PLEK SVM (Li et al., 2014) are the most widely used methods to predict the function of long non-coding RNA. The protein-coding potential of all identified full-length transcripts was predicted by screening the coding potential of transcripts using those tools. Firstly, PLEK and CNCI were used to predict coding potential according to full-length sequence characteristics. The sequences were then compared with the known protein databases using CPC2. Subsequently, the coding potential was predicted more accurately after mapping homologs to Pfam-A and Pfam-B databases. Non-coding transcripts identified by all software tools were considered lncRNAs. All remaining transcripts were considered to be protein-coding transcripts (mRNA). The open reading frames (ORFs) of all full-length transcripts of coding sequences (CDSs) were predicted using ANGLE V0.7 pipeline with default parameters (Shimizu et al., 2006). Transcription factor analysis was performed using the hmmsearch mapping technique against the animalTFDB 2.0 database (Zhang et al., 2015).

### Code Availability

The Iso-Seq library was constructed in accordance with the Iso-Seq protocol. Sequenced raw data were processed using SMRTlink v7.0. CCSs were generated from subreads with the parameters: min_length 50, max_drop_fraction 0.8, no_polish TRUE, min_zscore −9999.0, min_passes 1, min_predicted_accuracy 0.8, max_length 15000. CCSs were then classified into full length or non-full length reads. Full-length reads were isoform-level clustered using the algorithm: *n*^∗^log(*n*), followed by polishing using Arrow software with the parameters: hq_quiver_min_accuracy 0.99, bin_by_primer false, bin_size_kb 1, qv_trim_5p 100, qv_trim_3p 30. Nucleotide errors in consensus reads were corrected using Illumina RNAseq data using LoRDEC version 0.7 with the parameters: -k 23, s 3. Removal of redundancy of the corrected consensus reads was conducted using CD-HIT (-c 0.95, -T 6, -G 0, - aL 0.00, -aS 0.99, -AS30) to obtain final transcripts. Functional annotation was conducted based on seven databases.

Annotation was achieved using BLAST version 2.7.1 with *E*-value < 10^–5^ in the NCBI NT database, and Diamond version 0.8.36 software with *E*-value < 10^–5^ in the NCBI NR, KOG, Swiss-Prot and KEGG databases. HMMER v3.1 with hmmscan was used: -acc for the Pfam annotation.

For lncRNA prediction, CNCI version 2 and PLEK version 1.2 were used with default parameters. CPC2 version 0.1 based on the NCBI eukaryotes protein database was used with *E*-value < 10^–10^. A search of Pfam was performed using PfamScan version 1.6 with default parameters. Coding sequence prediction was performed using ANGEL version 2.4 with the parameter: -min_angel_aa_length 50. TF families in the animalTFDB 2.0 database were searched using the hmmsearch with default parameters. SSRs were identified using MISA version 1.0 with the default parameters: “1–12, 2–6, 3–5, 4–5, 5-4, and 6-5”^2^.

## Results and Discussion

### Sequencing and Data Processing

A total of 82.0 Gb of raw data were obtained from Iso-Seq sequencing using the PacBio SMRT sequencing method, containing 46.8 Gb nucleotides. Raw sequencing data were deposited into the NCBI SRA database with the accession number of PRJNA609058. After initial quality control by removal of the adaptor reads and subreads <50 bp, a total of 37,497,943 subreads (44.0 Gb nucleotides) were produced with a mean length of 1,174 bp (Supplementary Figure S1A,B). All subreads were thereafter used for CCS analysis. A total of 683,614 CCSs were produced (Supplementary Table S1 and Supplementary Figure S1C), in which 163,640 non-full length and 519,974 full-length subreads were produced, respectively. Among these full-length subreads, 485,881 (93.44%) were non-chimeric, with a mean length of 1,579 bp (Supplementary Table S1). After correction using next-generation sequenced short reads, 44,855 reads with a mean length of 1,595 bp were screened out (Supplementary Figure S1D). From the frequency of subreads prior to the removal of redundant data, the frequency of subreads of between 1,300–1,400 bp and 1,600–1,700 bp were the most prominent, indicating the relative abundance of expression of these transcripts. High-quality consensus reads were polished using Arrow software, with 69.00% of consensus reads ranging from 500 to 3000 bp (Supplementary Figure S1E). Finally, a total of 15,938 full-length non-redundant transcripts were produced after correction and redundancy removing by CD-HIT. Eighty percent of the full-length isoforms ranged from 500 to 4,000 bp, with only 984 isoforms longer than 4,000 bp (Supplementary Figure S1F). Only 8,959 transcripts were non-redundant (single copy) in the consensus reads. Significantly decreased numbers of non-redundant full-length transcripts exhibited good depth and high integrity using the SMRT long-read sequencing.

Transcriptomes comprehensively reflect the expressions of intracellular genes to reveal the physiological and biochemical processes at a molecular level. As a non-model insect without a known genomic sequence, NGS sequencing with *de novo* assembly is incapable of obtaining full-length transcripts. Iso-Seq has been used in *Agasicles hygrophila* to explore deep genetic information, in which 28,982 transcripts were obtained (Jia et al., 2018). This long-read sequencing resulted in fewer unigenes than the *de novo* transcriptome in brown citrus aphid (Shang et al., 2016), in which 44,199 unigenes were assembled. Aphids are cosmopolitan pests that attack a wide range of fruits and vegetables (Chaieb et al., 2018; Mohammed et al., 2018; Hong et al., 2019; Ullah et al., 2019; Ye et al., 2019; Shang et al., 2020). This was the first full-length transcriptomic sequencing in these multitudinous aphid pests.

### Gene Annotation

**FIGURE 1:**
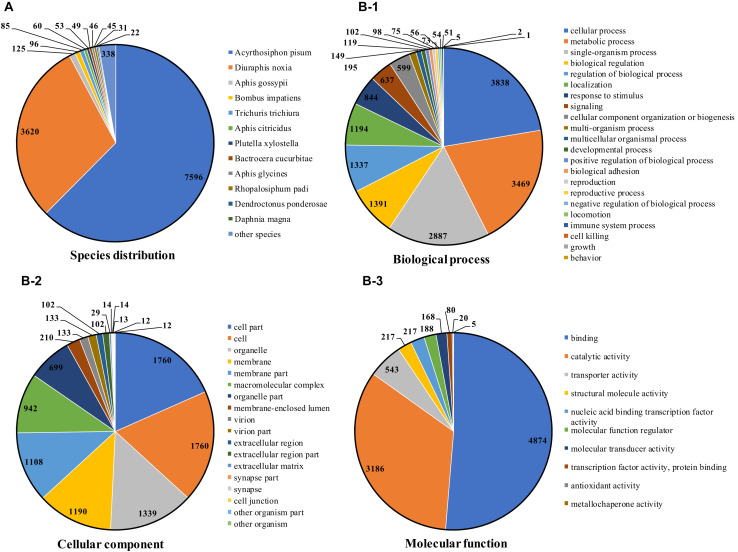
Species distribution annotated from the NCBI NR database **(A)**, and functional annotation using the GO database **(B)**.

The identified full-length transcripts were annotated in seven databases, and a total of 13,498 (84.69%) were annotated in at least one database, and 6,467 were annotated in all seven databases. Prediction and functional annotation of the coding transcripts were performed (Hong et al., 2020). Predictions indicated that 12,168 (76.35%), 12,581 (78.94%), 9,801 (61.49%), and 8,045 (50.48%) full-length transcripts were annotated in the NCBI non-redundant (NR), nucleotide sequence (NT), SwissProt and Pfam databases, respectively. According to the annotations in the NCBI NR database, the species were homologous in the majority of transcripts (11,216, 70.37%), which were annotated to two aphids, *Acyrthosiphon pisum* 7596 (47.66%) and *Diuraphis noxia* 3620 (22.71%) (Figure 1A). *Ac. pisum* is an important model organism for developmental and reproductive study in aphids, and the genome is also available (Richards et al., 2010). Similarly, the *D. noxia* genome is also available (Nicholson et al., 2015).

Regarding functional annotation, 8,045 (50.48%) transcripts were annotated against the Gene Ontology (GO) database, including 50 subcategories (Figure 1B). Similar to transcriptome analysis as described above, the most abundant GO terms in the biological processes category were “cellular process” (3,838), “metabolic process” (3,469), “single-organism process” (2,887). In the cellular component category, “cell” (1,760) and “cell part” (1,760) were the most common terms. “binding” (4,874) and “catalytic activity” (3,186) were the most common terms in the molecular function category.

**FIGURE 2:**
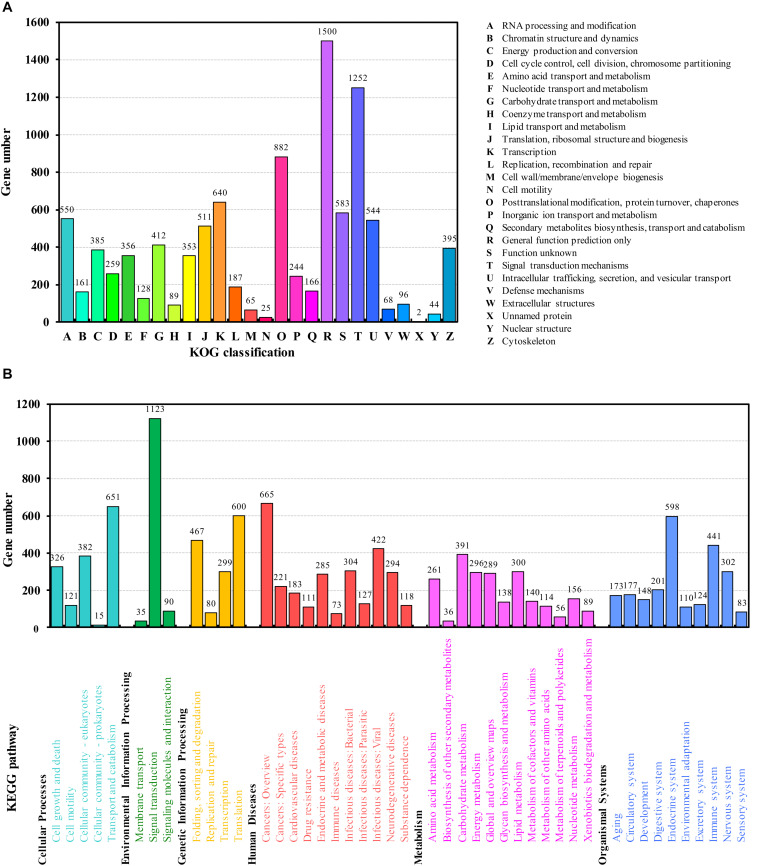
Functional annotation using the KOG **(A)** and KEGG databases **(B)**.

In euKaryotic Ortholog Groups (KOG) annotation analysis, 8,835 (55.43%) transcripts were classified into 26 subcategories (Figure 2A), the highest percentage of which were in general function. This is consistent with the results of *de novo* transcriptome of *A. citricidus* (Shang et al., 2016). A total of 11,372 transcripts were annotated in 349 pathways in the Kyoto Encyclopedia of Genes and Genomes (KEGG) database. These pathways were included in six level-one categories and 45 level-two subcategories (Figure 2B). The most prominent subcategory was “signal transduction.” The pathway analysis will help understand the high-level functions and utilize the biological systems, such as the cell, the organism, and the ecosystem (Kanehisa et al., 2008).

### Alternative Splicing and SSR Detection

**FIGURE 3:**
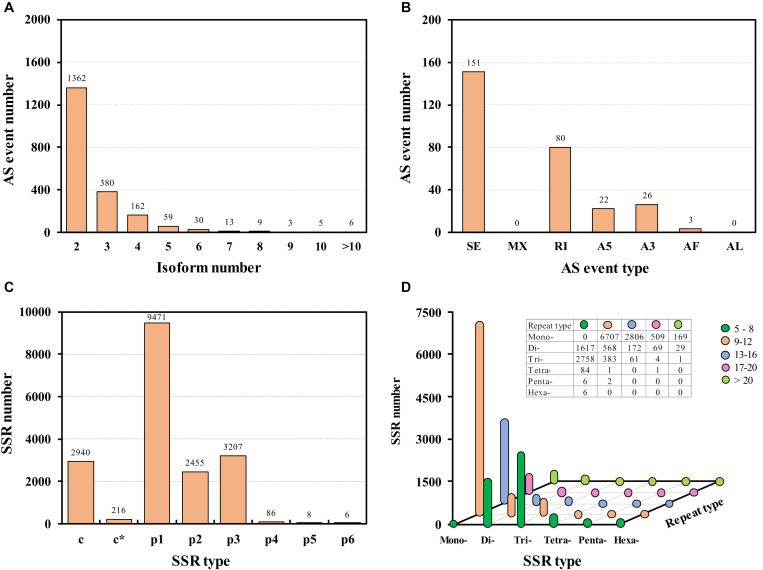
Alternative splicing (AS) events **(A,B)** and single sequence repeat (SSR) identification **(C,D)**. SE, skipping exon; MX, mutually exclusive exons; RI, retained intron; A5, alternative 5′ splice sites; A3, alternative 3′ splice sites; AF, alternative first exons; AL, alternative last exons; c, combined SSR; c*, combined and overlapping SSR; p1, mono-represent mononucleotide repeats; p2, di-represent dinucleotides repeats; p3, tri-represent trinucleotides repeats; p4, tetra-represent tetranucleotides repeats; p5, penta- represent pentanucleotides repeats; p6, hexa- represent hexanucleotides repeats.

RNA alternative splicing is widely existed in organisms. It occurs after a pre-mRNA transcript formed from template DNA, which results in multiple proteins from a single protein-coding gene. In this study, a total of 2,029 alternative splicing events were identified (Figure 3A). All the AS transcripts were deposited in figshare repository (Hong et al., 2020). Most of the AS events (1362, 67.13%) contained two isoforms. Since no reference genome is available in tea aphid, only 282 events including 1,480 transcripts were classified into five types of AS events in this SMRT sequencing (Figure 3B). The most prominent AS type was the skipping exon, but no mutually exclusive exons and alternative last exons AS were detected.

In order to study genetic diversity, evaluate quality and facilitate heredity studies, simple sequence repeats (SSRs) identified in the Iso-Seq library were analyzed. Excluding the complex SSRs, a total of 15,223 simple sequence repeats were detected (Figure 3C); all the SSRs predicted in *A. aurantii* were deposited in figshare (Hong et al., 2020). In these SSRs, 9,471 (62.22%) were mono-nucleotide repeats, and most of them were 9–12 repeats. Besides, there were 2,758 SSRs were Tri-nucleotide repeats with 5–8 repeats, and 1,617 SSRs were Di-nucleotide repeats with 5–8 repeats (Figure 3D). SSRs are co-dominant, hyper variable, neutral and reproducible molecular markers, and SSRs are now widely used in population genetic and conservation studies in many insects (Jing et al., 2012; Wang X. T. et al., 2019).

### LncRNA Prediction, Coding Sequence, and Transcription Factor Analysis

**FIGURE 4:**
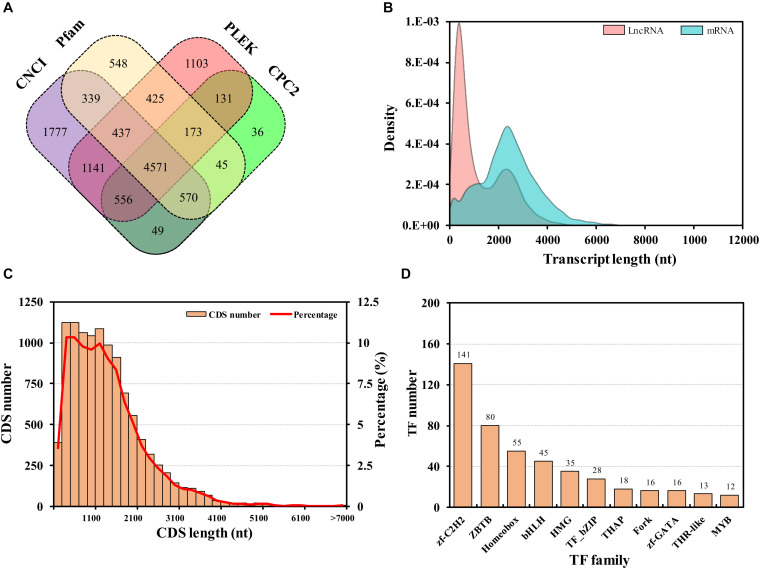
Long non-coding RNA (lncRNA) prediction **(A)**, expression density of lncRNAs and protein-coding RNAs (mRNAs) **(B)**, length distribution of protein-coding sequences (CDSs) **(C)**, transcription factor (TF) family statistics **(D)**. LncRNAs were predicted using CNCI, Pfam, PLEK, and CPC2 computational approaches.

Long non-coding RNAs constitute a major component of the transcriptome, which are defined as transcripts longer than 200 nt (nucleotides) in length without protein-coding potential (Yang et al., 2014). In this study, lncRNAs with a poly “A” tail were also sequenced using Iso-Seq. Following annotation, all full-length transcripts were predicted by mapping onto four databases. A total of 4,571 transcripts were identified as lncRNAs (Figure 4A). The exons of each lncRNA were not evaluated due to the lack of genome, while the expression densities of the lncRNAs and protein-coding RNAs were analyzed, which showed a high expression of short lncRNAs and low expression of long lncRNAs (Figure 4B). The lncRNAs identification was widely reported in eukaryote mammals (Kaushik et al., 2013; Zhan et al., 2016), plants (Lv et al., 2016), and microorganisms (Wang Z. et al., 2019). In insects, lncRNAs have been investigated in many species (Zhu et al., 2016), e.g., *Drosophila Melanogaster* (Wen et al., 2016), *Apis mellifera ligustica* (Chen D. et al., 2019), and *Nilaparvata lugens* (Chen M. Y. et al., 2019). In this study, only the lncRNAs with a poly “A” tailed were sequenced, so the number of the lncRNAs was less than that in other insects. For example, 8,096 putative lncRNAs were identified in *Plutella xylostella* (Wang et al., 2018).

Protein-coding transcripts were identified from the mRNA transcripts used for coding sequence (CDS) prediction by ANGEL. A total of 10,893 (95.83%) protein-coding transcripts were predicted (Figure 4C). The majority of these CDSs were shorter than 2,500 bp. In the present study, the number of coding transcripts was much less than other aphids (Richards et al., 2010; Nicholson et al., 2015). Transcription factor prediction in all unigenes was performed by Hmmsearch, from which 590 transcription factor transcripts were identified. The top three common families were zf-C2H2 (141), ZBTB (80), and Homeobox (55) (Figure 4D). The identification will be of convenience for further elucidation of transcriptional regulation.

In this study, we performed the full-length transcriptome sequencing using SMRT sequencing method, and 15,938 non-redundant full-length transcripts were obtained. All transcripts were annotated to seven databases, and 13,498 (84.45%) were functionally annotated. In addition, 4,571 (28.68%) transcripts were predicted as lncRNAs, and 590 were predicted as transcription factors. A total of 2,029 AS events and 15,223 simple sequence repeats were detected. This is the first Iso-Seq transcriptome of *A. aurantii*, and represents a significant increase in the known genetic information of *A. aurantii*. The data will assist in the future functional studies of genes involved in its development and reproduction, as well as the evolutionary studies of aphids.

## Data Availability Statement

The datasets presented in this study can be found in online repository: NCBI SRA database with the accession number PRJNA609058. The repository and the accession number can be found below: https://www.ncbi.nlm.nih.gov.

## Author Contributions

DW and FH conceived and designed the study, and analyzed the data. S-HM and X-YL contributed to the materials and samples. S-HM and FH contributed to the tables and figures. FH and DW drafted the manuscript. JN and JY revised the manuscript. All authors read and approved the final manuscript.

## Conflict of Interest

The authors declare that the research was conducted in the absence of any commercial or financial relationships that could be construed as a potential conflict of interest.

## Footnotes

1 https://github.com/comprna/SUPPA

2 http://pgrc.ipkgatersleben.de/misa/misa.html

## Supplementary Material

Supplementary Figure 1 Read length distribution during the filtering process. **(A)** Polymerase reads length distribution. **(B)** Subreads length distribution. **(C)** Circular consensus sequence (CCS) length distribution. **(D)** Full-length non-chimeric (FLNC) reads length distribution. **(E)** Consensus reads length distribution. **(F)** Gene length distribution.

Supplementary Table 1 Summary of reads after filtering.

## References

[B1] Alizadeh Kafeshani F. Rajabpour A. Aghajanzadeh S. Gholamian E. Farkhari M. (2018). Spatial distribution and sampling plans with fixed level of precision for citrus aphids (Hom., Aphididae) on two orange species. *J. Econ. Entomol.* 111 931–941. 10.1093/jee/tox380 29365154

[B2] Allan F. L. (1980). Transmission and properties of viruses isolated from *Carica papaya* in Nigeria. *J. Hortic. Sci.* 55 191–197. 10.1080/00221589.1980.11514922

[B3] Aslam S. Hamid F. S. Waheed A. Aslam N. Ahmed F. Zaman Q. (2015). Field evaluation of different chemical pesticides against aphid *Toxoptera aurantii* on different tea (*Camellia sinensis* L.) cuttings under high shade nursery. *Moroccan J. Chem.* 3 127–135.

[B4] Chaieb I. Zarrad K. Sellam R. Tayeb W. Ben Hammouda A. Laarif A. (2018). Chemical composition and aphicidal potential of *Citrus aurantium* peel essential oils. *Entomol. Gen.* 37 63–75. 10.1127/entomologia/2017/0317

[B5] Chen D. Chen H. Du Y. Zhou D. Geng S. Wang H. (2019). Genome-wide identification of long non-coding RNAs and their regulatory networks involved in *Apis mellifera ligustica* response to *Nosema ceranae* infection. *Insects* 10:245. 10.3390/insects10080245 31405016 PMC6723323

[B6] Chen M. Y. Ye W. Y. Xiao H. M. Li M. Z. Cao Z. H. Ye X. H. (2019). LncRNAs are potentially involved in the immune interaction between small brown planthopper and rice stripe virus. *J. Integr. Agric.* 18 2814–2822. 10.1016/s2095-3119(19)62569-4

[B7] Finn R. D. Coggill P. Eberhardt R. Y. Eddy S. R. Mistry J. Mitchell A. L. (2016). The Pfam protein families database: towards a more sustainable future. *Nucleic Acids Res.* 44 D279–D285. 10.1093/nar/gkv1344 26673716 PMC4702930

[B8] Gholamzadeh-Chitgar M. Pourmoradi S. (2017). An evaluation of the effect of botanical insecticide, palizin in comparison with chemical insecticide, imidacloprid on the black citrus aphid, *Toxoptera aurantii* Boyer de Fonscolombe and its natural enemy, *Aphidius colemani* Viereck. *J. Plant Prot. Res.* 57 101–106. 10.1515/jppr-2017-0013

[B9] Han B. Zhang Q. H. Byers J. A. (2012). Attraction of the tea aphid, *Toxoptera aurantii*, to combinations of volatiles and colors related to tea plants. *Entomol. Exp. Appl.* 144 258–269. 10.1111/j.1570-7458.2012.01303.x

[B10] Hong F. Han H.-L. Pu P. Wei D. Wang J. Liu Y. (2019). Effects of five host plant species on the life history and population growth parameters of *Myzus persicae* (Hemiptera: Aphididae). *J. Insect Sci.* 19:15. 10.1093/jisesa/iez094 31612945 PMC6792084

[B11] Hong F. Mo S. H. Lin X. Y. Niu J. Wei D. (2020). SMRT sequencing of the full-length transcriptome of the tea aphid, *Aphis aurantii*. *Figshare* 8:2197 10.6084/m9.figshare.5310025

[B12] Jia D. Wang Y. X. Liu Y. H. Hu J. Guo Y. Q. Gao L. L. (2018). SMRT sequencing of full-length transcriptome of flea beetle *Agasicles hygrophila* (Selman and Vogt). *Sci. Rep.* 8:8. 10.1038/s41598-018-20181-y 29396453 PMC5797098

[B13] Jing S. Liu B. Peng L. Peng X. Zhu L. Fu Q. (2012). Development and use of EST-SSR markers for assessing genetic diversity in the brown planthopper (*Nilaparvata lugens* Stal). *B. Entomol. Res.* 102 113–122. 10.1017/s0007485311000435 21896240

[B14] Kanehisa M. Araki M. Goto S. Hattori M. Hirakawa M. Itoh M. (2008). KEGG for linking genomes to life and the environment. *Nucleic Acids Res.* 36 D480–D484. 10.1093/nar/gkm882 18077471 PMC2238879

[B15] Kang Y. J. Yang D. C. Kong L. Hou M. Meng Y. Q. Wei L. P. (2017). CPC2: a fast and accurate coding potential calculator based on sequence intrinsic features. *Nucleic Acids Res.* 45 W12–W16. 10.1093/nar/gkx428 28521017 PMC5793834

[B16] Kaushik K. Leonard V. E. Shamsudheen K. V. Lalwani M. K. Jalali S. Patowary A. (2013). Dynamic expression of long non-coding RNAs (lncRNAs) in adult zebrafish. *PLoS One* 8:e83616. 10.1371/journal.pone.0083616 24391796 PMC3877055

[B17] Legeai F. Derrien T. (2015). Identification of long non-coding RNAs in insects genomes. *Curr. Opin. Insect. Sci.* 7 37–44. 10.1016/j.cois.2015.01.003 32846672

[B18] Li A. M. Zhang J. Y. Zhou Z. Y. (2014). PLEK: a tool for predicting long non-coding RNAs and messenger RNAs based on an improved k-mer scheme. *BMC Bioinform.* 15:311. 10.1186/1471-2105-15-311 25239089 PMC4177586

[B19] Li S. Hussain F. Unnithan G. C. Dong S. UlAbdin Z. Gu S. (2019). A long non-coding RNA regulates cadherin transcription and susceptibility to Bt toxin Cry1Ac in pink bollworm, *Pectinophora gossypiella*. *Pest. Biochem. Physiol.* 158 54–60. 10.1016/j.pestbp.2019.04.007 31378361

[B20] Li L. Wang M. Pokharel S. S. Li C. Parajulee M. N. Chen F. (2019). Effects of elevated CO2 on foliar soluble nutrients and functional components of tea, and population dynamics of tea aphid, *Toxoptera aurantii*. *Plant Physiol. Biochem.* 145 84–94. 10.1016/j.plaphy.2019.10.023 31675526

[B21] Li W. J. Song Y. J. Han H. L. Xu H. Q. Wei D. Smagghe G. (2020). Genome-wide analysis of long non-coding RNAs in adult tissues of the melon fly, *Zeugodacus cucurbitae* (Coquillett). *BMC Genomics* 21:600 10.1186/1471-2105-15-600 PMC7457495 32867696

[B22] Li W. Z. Godzik A. (2006). Cd-hit: a fast program for clustering and comparing large sets of protein or nucleotide sequences. *Bioinformatics* 22 1658–1659. 10.1093/bioinformatics/btl158 16731699

[B23] Lv Y. Liang Z. Ge M. Qi W. Zhang T. Lin F. (2016). Genome-wide identification and functional prediction of nitrogen-responsive intergenic and intronic long non-coding RNAs in maize (*Zea mays* L.). *BMC Genomics* 17:350 10.1186/1471-2105-15-350 PMC4865003 27169379

[B24] MacKenzie T. D. B. Arju I. Poirier R. Singh M. (2018). A genetic survey of pyrethroid insecticide resistance in aphids in new brunswick, Canada, with particular emphasis on aphids as vectors of Potato virus Y. *J. Econ. Entomol.* 111 1361–1368. 10.1093/jee/toy035 29474560

[B25] McGinnis S. Madden T. L. (2004). BLAST: at the core of a powerful and diverse set of sequence analysis tools. *Nucleic Acids Res.* 32 W20–W25. 10.1093/nar/gkh435 15215342 PMC441573

[B26] Mohammed A. A. A. H. Desneux N. Fan Y. Han P. Ali A. Song D. (2018). Impact of imidacloprid and natural enemies on cereal aphids: Integration or ecosystem service disruption? *Entomol. Gen.* 37 47–61. 10.1127/entomologia/2017/0471

[B27] Morandin C. Pulliainen U. Bos N. Schultner E. (2018). *De novo* transcriptome assembly and its annotation for the black ant *Formica fusca* at the larval stage. *Sci. Data* 5:180282. 10.1038/sdata.2018.282 30561435 PMC6298252

[B28] Nicholson S. J. Nickerson M. L. Dean M. Song Y. Hoyt P. R. Rhee H. (2015). The genome of *Diuraphis noxia*, a global aphid pest of small grains. *BMC Genomics* 16:429 10.1186/1471-2105-15-429 PMC4561433 26044338

[B29] Pinheiro P. V. Ghanim M. Alexander M. Rebelo A. R. Santos R. S. Orsburn B. C. (2017). Host plants indirectly influence plant virus transmission by altering gut cysteine protease activity of aphid vectors. *Mol. Cell. Proteom.* 16 S230–S243. 10.1074/mcp.M116.063495 27932519 PMC5393385

[B30] Piron P. G. M. de Haas M. C. Sonnemans M. A. H. M. (2019). The presence of *Aphis* (*Toxoptera*) *aurantii* (Homoptera: Aphididae) in the Netherlands. *Entomol. Ber.* 79 162–164.

[B31] Rao D. Capoor S. (1976). *Toxoptera aurantii*: an active vector of the Tristeza virus in India. *Indian J. Hortic.* 33 165–167.

[B32] Rhoads A. Au K. F. (2015). PacBio sequencing and its applications. *Genom. Proteom. Bioinf.* 13 278–289. 10.1016/j.gpb.2015.08.002 26542840 PMC4678779

[B33] Richards S. Gibbs R. A. Gerardo N. M. Moran N. Nakabachi A. Stern D. (2010). Genome sequence of the pea aphid *Acyrthosiphon pisum*. *PLoS Biol.* 8:e1000313 10.1371/journal.pone.1000313 PMC2826372 20186266

[B34] Sarac I. Ozdemir I. Karaca I. (2015). Ahids species in citrus orchards of Antalya province. *Munis Entomol. Zool.* 10 358–369.

[B35] Shang F. Ding B. Y. Xiong Y. Dou W. Wei D. Jiang H. B. (2016). Differential expression of genes in the alate and apterous morphs of the brown citrus aphid, *Toxoptera citricida*. *Sci. Rep.* 6:32099. 10.1038/srep32099 27577531 PMC5006003

[B36] Shang F. Ding B. Y. Ye C. Yang L. Chang T. Y. Xie J. (2020). Evaluation of a cuticle protein gene as a potential RNAi target in aphids. *Pest Manag. Sci.* 76 134–140. 10.1002/ps.5599 31461217

[B37] Sharma P. C. Grover A. Kahl G. (2007). Mining microsatellites in eukaryotic genomes. *Trends Biotechnol.* 25 490–498. 10.1016/j.tibtech.2007.07.013 17945369

[B38] Shimizu K. Adachi J. Muraoka Y. (2006). ANGLE: a sequencing errors resistant program for predicting protein coding regions in unfinished cDNA. *J. Bioinform. Comput. Biol.* 4 649–664. 10.1142/s0219720006002260 16960968

[B39] Sun L. Luo H. T. Bu D. C. Zhao G. G. Yu K. T. Zhang C. H. (2013). Utilizing sequence intrinsic composition to classify protein-coding and long non-coding transcripts. *Nucleic Acids Res.* 41:e166. 10.1093/nar/gkt646 23892401 PMC3783192

[B40] Thiel T. Michalek W. Varshney R. Graner A. (2003). Exploiting EST databases for the development and characterization of gene-derived SSR-markers in barley (*Hordeum vulgare* L.). *Theor. Appl. Genet.* 106 411–422. 10.1007/s00122-002-1031-0 12589540

[B41] Ullah F. Gul H. Desneux N. Gao X. Song D. (2019). Imidacloprid-induced hormesis effects on demographic traits of the melon aphid, *Aphis gossypii*. *Entomol. Gen.* 39 325–337. 10.1127/entomologia/2019/0892

[B42] Wang J. J. Tsai J. H. (2001). Development, survival and reproduction of black citrus aphid, *Toxoptera aurantii* (Hemiptera: Aphididae), as a function of temperature. *B. Entomol. Res.* 91 477–487. 11818043

[B43] Wang X. You X. T. Langer J. D. Hou J. Y. Rupprecht F. Vlatkovic I. (2019). Full-length transcriptome reconstruction reveals a large diversity of RNA and protein isoforms in rat hippocampus. *Nat. Commun.* 10:5009. 10.1038/s41467-019-13037-0 31676752 PMC6825209

[B44] Wang X. T. Zhang Y. J. Qiao L. Chen B. (2019). Comparative analyses of simple sequence repeats (SSRs) in 23 mosquito species genomes: Identification, characterization and distribution (Diptera: Culicidae). *Insect Sci.* 26 607–619. 10.1111/1744-7917.12577 29484820 PMC7379697

[B45] Wang Z. Jiang Y. Wu H. Xie X. Huang B. (2019). Genome-wide identification and functional prediction of long non-coding RNAs involved in the heat stress response in *Metarhizium robertsii*. *Front. Microbiol.* 10:2336. 10.3389/fmicb.2019.02336 31649657 PMC6794563

[B46] Wang Y. Xu T. He W. Shen X. Zhao Q. Bai J. (2018). Genome-wide identification and characterization of putative lncRNAs in the diamondback moth, *Plutella xylostella* (L.). *Genomics* 110 35–42. 10.1016/j.ygeno.2017.08.003 28789862

[B47] Wei D. Xu H. Q. Chen D. Zhang S. Y. Li W. J. Smagghe G. (2020). Genome-wide gene expression profiling of the melon fly, *Zeugodacus cucurbitae*, during thirteen life stages. *Sci. Data* 7:45 10.1038/s41597-020-0387-9 PMC7012831 32047161

[B48] Wen K. Yang L. Xiong T. Di C. Ma D. Wu M. (2016). Critical roles of long noncoding RNAs in *Drosophila* spermatogenesis. *Genome Res.* 26:1233. 10.1101/gr.199547.115 27516619 PMC5052038

[B49] Yang L. Froberg J. E. Lee J. T. (2014). Long noncoding RNAs: fresh perspectives into the RNA world. *Trends Biochem. Sci.* 39 35–43. 10.1016/j.tibs.2013.10.002 24290031 PMC3904784

[B50] Ye C. Jiang Y. D. An X. Yang L. Shang F. Niu J. (2019). Effects of RNAi-based silencing of chitin synthase gene on moulting and fecundity in pea aphids (*Acyrthosiphon pisum*). *Sci. Rep.* 9:3694. 10.1038/s41598-019-39837-4 30842508 PMC6403427

[B51] Yin Z. T. Zhang F. Smith J. Kuo R. Hou Z. C. (2019). Full-length transcriptome sequencing from multiple tissues of duck, *Anas platyrhynchos*. *Sci. Data* 6:9. 10.1038/s41597-019-0293-1 31754106 PMC6872741

[B52] Zekri N. Handaq N. El Caidi A. Zair T. Alaoui El Belghiti M. (2016). Insecticidal effect of *Mentha pulegium* L. and *Mentha suaveolens* Ehrh. hydrosols against a pest of citrus, *Toxoptera aurantii* (Aphididae). *Res. Chem. Intermed.* 42 1639–1649. 10.1007/s11164-015-2108-0

[B53] Zhan S. Y. Dong Y. Zhao W. Guo J. Z. Zhong T. Wang L. J. (2016). Genome-wide identification and characterization of long non-coding RNAs in developmental skeletal muscle of fetal goat. *BMC Genomics* 17:666 10.1186/1471-2105-15-666 PMC4994410 27550073

[B54] Zhang H. M. Liu T. Liu C. J. Song S. Y. Zhang X. T. Liu W. (2015). AnimalTFDB 2.0: a resource for expression, prediction and functional study of animal transcription factors. *Nucleic Acids Res.* 43 D76–D81. 10.1093/nar/gku887 25262351 PMC4384004

[B55] Zhang Q. L. Wang F. Guo J. Deng X. Y. Chen J. Y. Lin L. B. (2018). Characterization of ladybird *Henosepilachna vigintioctopunctata* transcriptomes across various life stages. *Sci. Data* 5:180093. 10.1038/sdata.2018.93 29870033 PMC5987669

[B56] Zhou Y. Y. Zhou B. Pache L. Chang M. Khodabakhshi A. H. Tanaseichuk O. (2019). Metascape provides a biologist-oriented resource for the analysis of systems-level datasets. *Nat. Commun.* 10:1523. 10.1038/s41467-019-09234-6 30944313 PMC6447622

[B57] Zhu B. Liang P. Gao X. (2016). Long noncoding RNAs (lncRNAs) and their research advances in entomology. *Acta Entomol. Sin.* 59 1272–1281.

