# Supplementary material for: The PacBio Full-Length Transcriptome of the Tea Aphid as a Reference Resource
Source: Front Genet. 2020 Nov 12;11:558394. doi: 10.3389/fgene.2020.558394 (PMC7693467; doi:10.3389/fgene.2020.558394)
Supplement: Supplementary Figure 1 — Read length distribution during the filtering process. (A) Polymerase reads length distribution. (B) Subreads length distribution. (C) Circular consensus sequence (CCS) length distribution. (D) Full-length non-chimeric (FLNC) reads length distribution. (E) Consensus reads length distribution. (F) Gene length distribution. [file Data_Sheet_1.docx]

Supplementary Material

# Supplementary Tables

**Table S1.** Summary of reads after filtering.

| Classification | Number | Minimum length | Maximum length | Mean length | N50 |
| --- | --- | --- | --- | --- | --- |
| CCS | 683,614 | 52 | 14,989 | 1,845 | 2,495 |
| FLNC read | 485,881 | 50 | 14,445 | 1,579 | 2,207 |
| Consensus read | 44,855 | 53 | 11,495 | 1,595 | 2,378 |
| Unigene | 15,938 | 60 | 11,502 | 2,039 | 2,664 |

CCS: circular consensus sequences; FLNC read: full-length non-chimeric read.

# Supplementary Figures

**Figure S1**. Reads length distribution during the filtering process. **A**, polymerase reads length distribution; **B**, subreads length distribution; **C**, circular consensus sequence (CCS) length distribution; **D**, full-length non-chimeric (FLNC) read length distribution; **E**, consensus read length distribution; **F**, gene length distribution.
